# Schizophrenia: jinn, magic or disease? Experiences of family caregivers of patients with schizophrenia in Baloch ethnicity

**DOI:** 10.1186/s12888-023-05332-4

**Published:** 2023-11-13

**Authors:** Fatemeh Darban, Enayatollah Safarzai, Sakineh Sabzevari, Nastaran Heydarikhayat

**Affiliations:** 1 Department of Nursing, School of Medicine, Iranshahr University of Medical Sciences, Iranshahr, Iran; 2 Department of Nursing, Ali-Ebne-Abitaleb Hospital, Zahedan University of Medical Sciences, Zahedan, Iran; 3 Nursing Research Center, Kerman University of Medical Sciences, Kerman, Iran; 4 Department of Nursing, School of Medicine, Iranshahr University of Medical Sciences, Iranshahr, Iran

**Keywords:** Qualitative study, Schizophrenic disorders, Culture, Superstitions, Caregiver

## Abstract

**Background:**

Cultural and religious beliefs are effective on people’s attitudes towards schizophrenia and their help-seeking behaviors. This study aimed to explain the experiences of family caregivers of patients with schizophrenia in Baloch ethnicity.

**Methods:**

This is a qualitative study with conventional content analysis approach. Purposive sampling was used and 21 participants, including family caregiver for patients with schizophrenia, a psychologist, a prayer-writer, and a normal person were interviewed in Sistan and Balochistan province in the southeast of Iran. Qualitative data were analyzed by Granheim and Lundman method.

**Results:**

One main theme, three categories, and 10 Sub-categories were extracted from analysis of interviews. “Immersion in the cultural beliefs” was the main theme of the study with categories of “Belief in the superstitious and supernatural nature of the psychological disease”, “Superstitious beliefs, an attempt to free the patient”, and “Conflict between cultural beliefs and science”.

**Conclusions:**

Help-seeking behaviors of family caregivers in Baloch ethnicity are influenced by their religious, superstitious, and cultural beliefs. Psycho-education should be part of all mental health education programs in these communities, as delays in treatment worsen the prognosis of people with schizophrenia. Training the medical staff to consider the culture, religion and therapeutic preferences of the Baloch people can be effective in advancing the goals. In addition, local influencers should stress the importance of health care alongside harmless local remedies.

## Background

Schizophrenia is one of the most devastating chronic mental disorders and generally begins before the age of 21 and will continue through life [1]. Schizophrenia is characterized by variations from normal in two or more of the five areas of delusions, hallucinations, disturbed thinking (speech), profoundly exacerbated or abnormal motor behavior, and negative symptoms [2]. The prevalence of schizophrenia is generally around 4.6-5 per 1000 people and is considered to be the fourth leading cause of disability in the world [3, 4]. Schizophrenia causes a devastating social, health and economic burden to individuals, families and society [5]. Despite the available evidence on the impact of schizophrenia on family members, it is necessary to take into account the culture and traditions of each country and ethnicity, as culturally adapted interventions are more effective and acceptable [6]. Culture and mental health are two completely interrelated concepts [7]. Schizophrenia is a culturally dependent disease and even its manifestations are influenced by culture [8]. Cultural beliefs, poor knowledge, religious beliefs, race, language, misconceptions and social beliefs are among the factors that influence people’s attitudes towards mental illness [9, 10]. Religious ideologies, superstitions and social beliefs can influence how people identify the cause and treatment of mental illness [11].

The Baloch people live in Sistan and Balochistan province in southeastern Iran and have important characteristics such as language, religion and culture. Most Baloch are Muslim, Sunni and speak Balochi. The Baloch ethnicity is a traditional society with tribal identity and male dominance and with extended families. People’s behavior is strongly mixed with traditional, cultural and religious beliefs and tribal life, hard work, patience and adaptability are the main characteristics of its inhabitants [12–14].

Baloch ethnic culture is profoundly established in Islamic values and emphasizes the significance of family harmony and caring for powerless relatives [13, 15]; family members have a close relationship with each other, with care and a shared responsibility towards one another [16]. Baloch ethnicity has similarities in its religious beliefs and teaching with Arab and neighboring countries like Pakistan and India [17]. Lim et al. (2018) state that cultural anxiety has arisen in Muslim societies that attribute the root of psychiatric disorders to supernatural factors such as the evil eye and jinn possession [18]. Various studies in Arab Muslim countries showed that the general public considers supernatural factors such as jinn and black magic to be involved in the etiology of psychiatric disorders [19, 20]. These beliefs about the cause of schizophrenia are the main forces that lead the public to change treatment methods and delay professional medical treatment, thus changing the outcome of the disease [21]. The experience of mental health help-seeking varies by contexts and populations [22]. Seeking help from faith healers is therefore often the first step in managing schizophrenia. This leads to significant under-diagnosis and long delays in getting medical help to those who require it [17]. Psychological interventions for people with schizophrenia in Pakistan are influenced by factors such as poor access to psychiatric care, scarcity of specialists and resources, intangible beliefs and referral to traditional healers [23, 24]. Direct medical costs associated with mental illness, low educational attainment, poor knowledge of mental health, stigma, perceived ineffectiveness of mental health interventions, and fear of being officially classified as having a mental illness have been cited as reasons for the delay in first-onset psychosis in South Korea, China, and the United States [25–28].

Despite the unique characteristics of Baloch ethnicity, no study has been found to access their beliefs about psychiatric disorders and examine the help-seeking behavior of these people for a person with schizophrenic behavior. In addition, information from the psychiatric centers regarding the population of 2,775,14 people in the vast Sistan-Balochistan province at the time of this survey is: There is one psychiatric hospital in Zahedan, two psychiatric wards in Iranshahr and Zabol, but no hospitals or psychiatric wards in other cities. Patients’ access to medical centers in the province and neighboring provinces is limited due to long distances. Less than a third of people with schizophrenia have access to treatment in centers with low resources. Interventions are also limited due to lack of infrastructure, social services and funding [29]. Therefore, it seems necessary to conduct an in-depth study with a qualitative approach to obtain the beliefs and practices of the Baloch people who take care of a schizophrenic patient in their family. This method can show the nature of the phenomenon and its formation in the natural context. Different behaviors of people are culturally related, and cultural context should be considered when interpreting the meaning of a particular behavior [30, 31]. In addition, people’s perceptions and judgments about mental illness and patient care are influenced by culture, resulting in different caregiving experiences [32]. The family’s role in the development of the patient’s mental health is critical because the family is in a unique position due to prolonged contact and interaction with the patient and members of the care team and various aspects of care duties [30, 33]. The researchers therefore decided to use a qualitative research approach to conduct a study to explain the experiences of Baloch families with members suffering from schizophrenia.

## Methods

### Design and setting

This is a qualitative study with a conventional qualitative content analysis approach. Content analysis as a research method and scientific tool can provide new insights into phenomena, improve researchers’ understanding of phenomena, and identify operational strategies [30]. This study was conducted from January to July 2022. The setting of this study was Sistan and Balochistan, the second largest province in Iran after Kerman [34]. With a population of more than 2,775,014 million, this province is bordered by South Khorasan province and Afghanistan to the north, Pakistan and Afghanistan to the east, the Oman Sea to the south, and the provinces of Kerman and Hormozgan to the west. The inhabitants of this province have two ethnicities, the Baloch and the Sistani. The Baloch speak the Balochi dialect and are Sunni Muslims [35]. Sistan and Balochistan is the poorest of Iran’s 31 provinces with a Human Development Index (HDI) of 0.688 [34]. Education in the province is also facing problems, with media reporting “shocking statistics on education deprivation in Sistan and Balochistan” and “lack of basic facilities for education in schools of this province.“ People in this province are also deprived of sports and entertainment facilities. The lack of medical care and treatment in the state has been the subject of media attention [36].

### Participants and sampling

Participants were family caregivers of patients with schizophrenia who met the following inclusion criteria: be in good physical and mental conditions, be responsible for the direct care of the patient, living with patient in the same home, be at least 18 years of age [25, 29], caring for patient at least one year since the diagnosis of schizophrenia [37]. Purposive sampling was used. To consider maximum transferability, people were selected from different social, educational, economic and residential levels.

Sampling continued until data saturation was reached. In this study, data was saturated with 18 interviews but for more assurance, the interview was continued until 21 interviews. The researcher approached one of the psychiatric centers in Sistan and Balochistan province and started sampling. The researcher referred to the medical records departments of this hospital and contacted the patient’s family members from the address and telephone number recorded in the medical file and made the necessary arrangements to conduct the interview.

### Data collection

Initially, the researcher contacted each participant by telephone and, if eligible, obtained their consent, and set a convenient time and place for an interview based on the participant’s preference. Data were collected using semi-in-depth and semi structured, face-to-face interviews. To conduct the interviews, a list of guide questions was first prepared according to the research objective. For this purpose, a meeting was held among the members of the research team. All members discussed the questions, and finally the most important and relevant questions were selected. During the various interviews, questions were added to this list according to the data obtained.

The main framework of the interview guide was questions about the experiences of caregivers caring for a person with schizophrenia and their attitudes towards help-seeking. Some of the main questions were as follows: 1- Please tell us about your experience of caring for a person with schizophrenia. 2- What do you consider to be the root cause of your patient’s current problem? 3- What steps did you take when you noticed the symptoms? 4- What local interventions are used in your ethnicity to treat people with schizophrenia? According to the answers of the participants, Probing questions were asked to gain a better understanding of the participants’ experience including “What do you mean?“ or “explain more”, were also used. According to the participants’ preference, the interviews were conducted at the participant’s home or in a room in a psychiatric center that was quiet. All interviews were conducted in Balochi dialect by the first author (F.D.) and translated into Farsi prior to analysis. She has a doctorate in psychiatric nursing and is a Baloch.

The time of the interviews was 40–100 min. All interviews were recorded with the consent of the participants. Immediately after the interview, after listening several times, the transcript was typed word by word. The interview process was evaluated by the second and third authors. In addition to interviews, field notes were also used in the data collection process.

### Data analysis

MAXQDA version 20 software was used for frequent comparison of different data and ease of data organization. Concurrent with the data collection, the analysis was done using the conventional content analysis method and according to the steps proposed by Graneheim and Lundman. In this method, semantic unit, summarization, code, category and theme are important. In this method, data analysis begins by repeatedly reading the text to become immersed in them and find a general sense; then the texts are read word by word to extract the codes. After that, the codes are classified into classes based on similarities and differences, and at the end, evidence from the text of the data is quoted for each concept [38].

After reviewing the text of the interviews several times, it was divided into constitutive semantic units and the smallest meaningful units. The codes were then re-read to put into subcategories based on semantic similarity and then the main categories were formed. Finally, the main classes were set up according to an abstract concept (theme). All authors have reviewed the extracted codes and categories [30].

### Trustworthiness

Lincoln and Guba’s four criteria (credibility, dependability, transferability, and conformability) were used to ensure the rigor of the findings [39]. For the transferability of the findings, an effort was made to diversify participants in terms of education level, family relationship with the patient, economic status and social class, which also contributed to the parts that make the maximum variation. As for the reliability of the findings (dependability), all phases of the study were recorded and reported. For the credibility of the findings, all extracted codes of each interview were checked with the interviewee (member check) and modified if necessary. To confirm the findings, an external faculty member (faculty check) and all other authors of this study (peer check) checked all the interviews, codes and extracted categories [30, 40].

### Ethical considerations

The ethics committee of Iranshahr University of Medical Sciences approved this study with code IR.IRSHUMS.REC.1400.014. Obtaining informed consent from the participants and assuring them of their anonymity and confidentiality of data was another ethical consideration. Participants chose the time, place and duration of the interview. The audio files were stored in a safe place in an encrypted file on the computer by the first author. The participants were free to withdraw from the study at any stage of the research. In addition, this data collection was done by one of the Baloch authors to protect the dignity of patients and their families and to avoid stigma from neighbors when a researcher visited their homes. The first author was of Baloch ethnicity, and because she wore Baloch clothes, people thought she was a guest. On the other hand, there was a sense of unity and connection among caregivers.

## Results

### Descriptive findings

13 women and 8 men, all Baloch and with an average age of 41 years, participated in this study. The education level of most of them was diploma. More details are provided in Table 1.

### Qualitative findings

After analysis, 428 primary codes emerged. Following integration, 312 similar codes, 10 sub-categories, 3 categories and one theme were extracted, Table 2. “Immersion of the family in the cultural beliefs” was the main theme of the study with categories of “Belief in the superstitious and supernatural nature of the psychological disease”, “Superstitious beliefs, an attempt to free the patient”, and “Conflict between cultural beliefs and science”.

**Table 1:** Demographical characteristics of family caregivers of patients with schizophrenia

#	Participants	Age (Y^*^)	Gender	Educational level	Employment	The duration of the disease (Y)
1	Sister	39	Female	Elementary	Unemployed	8
2	Spouse	40	Male	Elementary	Paid employment	22
3	Sister	30	Female	Diploma	Paid employment	5
4	Brother	33	Male	Elementary	Paid employment	9
5	Mother	45	Male	Elementary	Unemployed	5
6	Spouse	31	Male	Elementary	Paid employment	9
7	Mother	65	Male	Elementary	Unemployed	8
8	Spouse	43	Male	BSc^υ^	Full time employment	10
9	Child	32	Male	MSC^†^	Full time employment	18
10	Spouse	50	Female	Elementary	Paid employment	15
11	Father	51	Male	BSc	Paid employment	5
12	Child	26	Female	BSc	Unemployed	20
13	Child	27	Female	Diploma	Unemployed	15
14	Father	55	Male	Elementary	Paid employment	5
15	Mother	48	Female	Elementary	Unemployed	4
16	Uncle	35	Male	BSc	Full time employment	6
17	Brother	40	Male	Elementary	Paid employment	8
18	Prayer writer	46	Male	Diploma	Prayer writer	
19	Recovered patient	46	Female	Elementary	Unemployed	
20	Psychologist	38	Female	PhD	Full time employment	
21	Normal person in the community	30	Female	MSc	Full time employment	

*Y: year, υ: Bachelor degree, †: Master of Science

**Table 2:** Extracted sub-categories, categories and theme based on the experiences of family caregivers of patients with schizophrenia in Baloch ethnicity

Theme	Categories	Sub-categories
**Immersion in the cultural beliefs**	Belief in the superstitious and supernatural nature of the psychological disease	Possessed by jinn
		Delusion, the manifestation of bewitchment
		Mental illness, conspiracy of those around
		Sinking into the superstitions
		Cultural erroneous believability
	Superstitious beliefs, an attempt to free the patient	Prayer writers as local experts
		Spell breakers, earthly saviors of patients
		Superstitions, a way to escape stigma
	Conflict between cultural beliefs and science	Adherence to the treatment, the victim of superstition
		Religious beliefs parallel with scientific therapy

## Theme: immersion in the cultural beliefs

It reflects the impacts of cultural beliefs and superstition on caregivers’ view and decision for family member with psychological disease. In addition to caregivers and patients, some members of the medical team such as nurses or paramedic/nurse assistants and people around them, share similar beliefs.

### Category 1: belief in the supernatural nature of the psychological disease

The initial symptoms of the disease were interpreted as abnormal behaviors by the family members. By comparing their patient’s symptoms to physical illnesses that are more tangible and objective in nature, the interviewees had an abnormal perception of their patient’s psychiatric symptoms and considered the patient’s actions and behavior unusual and felt that changes were occurring. Many disease symptoms and the participants’ interpretations of these symptoms are strongly influenced by the cultural context and religious cultural beliefs of that province; similarly, hallucinations are interpreted as insanity or delusion, and patient’s misbehavior as magic. Altered thoughts and feelings, confusion, and irritability are considered abnormal behaviors and psychotic disorders. People called the patients with schizophrenia “Ganok”, which means “crazy” in the Baloch language. There are three sub-categories in this category, including “Possessed by jinn”, “Delusion, the manifestation of bewitchment”, “Cultural erroneous believability”. The main concepts in these subcategories focus on the cultural beliefs about the causes of schizophrenia, non-medical treatments, and the challenges and conflicts between medical and non-medical treatments in the Baloch cultural context.

#### Sub-category 1: possessed by jinn

Hallucination was one of the symptoms that the participants encountered and mentioned as strange and unusual behavior. The content of the patient’s hallucinations was influenced by the local culture of the province and was formed based on local expectations and meanings, and most patients had delusions about jinn, angels (Parry in the local language), etc. In the native culture of the Sistan and Balochistan province, the patient’s hallucinations are called visions of jinn. Seeing the jinn and hearing the voice of the jinn are terms so ingrained that even in psychiatric centers they are used to take into account the psychiatric history of patients and their families. They asked if the patient had ever seen a jinn.

“*He saw the jinn and he smelled the jinn, he also told us that it smelled bad, and sometimes he pulled the blanket over his head to keep the smell out. Sometimes he talks to himself under the blanket. At times we thought he was having a physical fight with someone and it was as if he was talking to a group and said, “Leave me alone, and don’t beat me” (p2).*

*Another caregiver said*

“He saw a jinn and we saw him talking to someone. He said that, the jinn appeared to me in the form of a strong man. I think he told us the truth and he saw it, but we didn’t see it (p10).

#### Sub-category 2: delusion, the manifestation of bewitchment

Delusion was one of the symptoms that the participants pointed to as a sign of the patient becoming bewitched when they first encountered it. The most important perceptions of caregivers with regard to patient’s delusions include delusion of persecution, grandiosity, being spellbound, and afraid of magic as well as fear of becoming poisoned. Being paranoid in a marital relationship was also common among married couples. This means that the mentally ill woman or man suspected his or her spouse of being unfaithful. These delusional beliefs were influenced by the culture and religion of this province. Magic, ethnicity and tribe, religious beliefs, power, shame, masculinity and honor were important variables that influence the culture of this province and have a major impact on patients’ delusions.

*“She was bewitched, she didn’t eat anything, she said there was poison in her food that would kill her, she was very upset, she didn’t even drink water and she went to drink water from the tap in the yard, which was hot and salty” (p3).*

Another caregiver talked about black magic as:

“My son is a marine engineer. He did a training course on a ship that travelled all over the world. He went to Europe, Africa and America. He was in a bad mood when he was doing the training course. He called and told us that, we have a marine engineer who is black and from Kenya. He has performed black magic on me and now I’m not well” (p7).

#### Sub-category 3: sinking into the superstitions

Attributing the symptoms of disease to supernatural factors caused families to sink into the superstition. Many believe that the disease is caused by the evil eye. They believe that children should not leave the house in the evening because they will be possessed by demons, or that ashes should not be kicked up in the evening and at night, or that the hot water should not be poured outside because it may be spilled on the jinn’s children and make them angry.

One of the participants expressed the superstition as:

*“They told his brother that, you should bring a sheep and sacrifice it to give blood to the jinn. His poor brother gave a lot of money to prayer writers in the hope that she would be cured. But it had no effect, it made his even worse. Finally, the prayer writer said because the jinn married him and won’t let him go, he won’t get better” (p10).*

In addition, participants believed that one should not go to the cemetery or ruins at night and that the baby swaddling should not be white because the jinns like it and will play with the baby and this will cause harm.

#### Sub-category 4: mental illness, conspiracy of those around

The confusion in the families due to the onset and worsening of the patient’s symptoms led them to suspect others as the cause of the patients’ illness. Through misinterpretation, the patient’s family members attributed these symptoms to causes such as witchcraft. Most of the participants said that when they saw the patient’s condition, they suspected that someone they knew and the people around them had bewitched the patient into suffering these symptoms, because they thought it was more than a disease.

*“My husband’s financial situation was very good. When he got sick, we thought that someone around him wanted to hurt him financially and put a spell on him. We had a tenant who said that he was clearly being cursed” (p8).*

Another participant said that:

*“Because my father had a lot of land, my first thought was that my relatives and cousins ​​had bewitched him for the land so that he would be possessed and the land would remain for them, and now the land is left for them and they have reached their goal” (p1).“*

#### Sub-category 5: cultural erroneous believability

The early onset of the disease with hallucinations and delusions, without other obvious symptoms, made some participants compare the patient’s states and behavior with reality and cultural beliefs and accept them as true. Delusions of magic and persecution were among the plausible symptoms for the participants. Belief in the patient’s delusions increased when the patient’s first symptoms occurred outside the home environment or those who did not live with the patient believed more in the patient’s delusions. Believing the patient’s delusions led to various reactions, such as discomfort, blaming the accused person, and even negotiations between the two clans.

“*My husband came back from Yazd (a city in Iran) and said that, your brothers sent someone to kill me. My father and brothers were very upset about this. They said that if something bad happens to him, his family and your children will think that your brother committed the crime. So they informed his family of this matter, and asked why he was slandering them, and sent a message to his brother that we should negotiate” (p4).*

Another participant said that:

*“When he was discharged from hospital, he was much better; that’s why we thought he must be telling the truth, and black magic must have made him sick “(p6).*

### Category 2: superstitious beliefs, an attempt to free the patient

This category reflects the effort of the caregivers to save patients from unpleasant symptoms. Because people believe in non-scientific reasons for the roots of disease, they try to use cultural methods for treatment of the psychological disorders. This category has two sub-categories including “Prayer writers as local experts” and “Spell breakers, earthly saviors of patients”.

#### Sub-category 1: prayer writers as local experts

According to the participants’ statements, the cultural and religious beliefs about jinn and magic made the families of the patients imagine the symptoms of the patients as supernatural and seek non-medical treatments. Referral to various prayer writers, unprincipled treatments and high costs makes families wander if their efforts were fruitless, which led to the deterioration and severity of the patient’s symptoms. Before going to the doctor, they used to refer to prayer writers to check the symptoms; Asking for help from prayer writers to remove the symptoms mostly occurred by families who thought that the symptoms were caused by demons or by bewitching the patient. Caregivers spend a lot of time visiting prayer writers, so sometimes frequent visits to prayer writers worsened the patient’s symptoms and delayed the treatment process. In some cases, consultation with prayer writers was so expensive. Prayer writers endorse the supernatural nature of the patient’s symptoms, and knowing that charms and prayers are ineffective for patients with schizophrenia, they prescribed these treatments to patients and their families in the hope that the patient will recover. People are used to wasting a lot of money and time waiting for these unqualified therapists; they followed the medicine man’s instructions.

*“We took her to different prayer-writers several times, they said that she was bewitched, and they gave amulets and prayers to get rid of her symptoms” (p.15).*

Participant 20 said:

*“Without exaggeration, when I asked all the families of our patients, they definitely mentioned that before they went to a psychiatrist, they went to a prayer writer at least once. It is really a disaster (P.20).*

Depending on the type and severity of symptoms, prayer writers offer various local treatments such as harmala burning (Esfand burning in native language), charms, using smoothing plants such as cloves and roses, blessing the water (by reading prayers and the words on it(.

One of the participants who was also a prayer writer said:

“First, I pray on their heads and blow on their faces, for a period of 7 to 10 days, I give them amulets to hang on the patient’s neck or arm. In addition, sometimes I give them some herbal medicine; Herbs such as gilli flowers and roses are recommended for calming. There are always people who believe in us and spend big bucks, even though we know these things are ineffective and a form of brainwashing for patients and their loved ones” (p18).

#### Sub-category 2: spell breakers, earthly saviors of patients

The people of this province believe that medical treatments do not help and those who invalidate spells are the saviors of people. Participants reported long queues for some spell breakers, waste of time and money, and complete trust in these local persons. To free their patients from psychological symptoms and become infamous among the neighbors, people did strange and illogical things at the request of spell breakers.

One of the participants said:

*“The spell breaker said that to break the spell, you should bring a white hen to kill it in the evening and its blood will break the spell. We paid a lot and he told me to come tomorrow and get the spell breaker. There were many crooked lines written on folded papers and he said to put one in a fish’s mouth and sew up the fish’s mouth with thread and throw it into the river”.*

#### Sub-category 3: superstitions, a way to escape stigma

According to the participants’ statements, the stigma and negative attitude of the people of this province towards psychiatric disorders caused families to try to attribute the symptoms of the disease to jinn or magic. Attributing symptoms of disease to jinn or magic would reduce the psychological pressure of families. Because in the culture of the region, conflict between jinn and magic was more accepted than psychiatric disorders.

*People call them crazy, some call them psychopaths (mad), but when they realize that they’ve become jinn or they are bewitched, they know that it’s not their fault and they feel sorry for them” (p17).*

### Category 3: conflict between cultural beliefs and science

One of the main barriers that families faced was the belief in supernatural effects such as jinn and magic on the development and treatment of psychological diseases. Being caught in the trap of superstitions had many negative consequences for the families and patients.

This category consists of two subcategories including: “Adherence to the treatment, the victim of superstition” and” Religious beliefs reinforcing medical treatments”. Religious beliefs parallel therapy with scientific therapy.

#### Sub-category 1: adherence to the treatment, the victim of superstition

The cultural beliefs that prevail in Sistan and Balochistan province cause a negative interpretation of symptoms and treatment of the mentally ill patients. Participants attributed the symptoms and cause of the disease to supernatural factors and consider demons and magic as the cause of the disease in the family member. The same issue has made the treatment process from the time of diagnosis to hospitalization, and also the follow-up after discharge, subject to superstitions, which leads patients/families to stop the treatment and turn to non-scientific treatments. There is a gap between the start of treatment and the effect of the drugs and different treatment methods that need to be given time, this point causes people to distrust the treatment and stop the drugs. On the other hand, after being discharged from the hospital, the lack of strict supervision of the family on the use of drugs, which led to the recurrence of symptoms, was attributed to the ineffectiveness of the treatment.

One of the participants said:

*“We have a patient to whom the jinn appeared, he saw the jinn; when he saw the jinn twice, it affected his nerves and heart, and he has had to go to a psychiatrist and to a mullah (a prayer writer) to cure its effects. Some people are so strongly affected by magic that no medical treatment can help them” (p21).*

The effect of superstitious beliefs on non-compliance with treatment was expressed as follows in the speech of one of the participants:

*“After hospitalization, my sister’s symptoms improved, but at home, they didn’t always give her medication regularly. When the symptoms returned, everyone told us that hospitalizing her will only damage the reputation of the family, and the neighbors will say that they have a mentally ill daughter. These drugs only damage her brain and have no effect” (P.1).*

#### Sub-category 2: religious beliefs parallel with scientific therapy

The cultural and religious context of Sistan and Balochistan province has caused religious teachings to strongly affect issues related to health and illness. Religious beliefs, the highlighted role of a religious person in people’s lives, and people’s belief in the influence of religious schools in raising children have all caused caregivers to seek help from the religious aspect of their culture to calm the agitated and mentally ill patients. Reciting religious books at home, praying and always mentioning God are ways to keep extraterrestrial beings away from the home and achieve peace in these people. The trust of the people of this province in religious scholars and clerics led some participants to take their patients to these scholars when the symptoms appeared, and then go to the psychiatrist after confirming their statement that the symptoms were psychiatric. There were some patients that were also referred to psychiatrists by these clergymen (Maulavi in native language).

One participant said:

*“Yes, we took him to Maulavi…; when we took him, he said take him to a hospital. These are from his mind, and for this problem, I cannot do anything for him, and it will not be cured with amulets“(p13).*

One of the local treatments that is rooted in the religious beliefs in Sistan and Balochistan is Ruqyah treatment. This program includes the recitation of Quranic verses to calm the patient and to keep the jinn away from the mental patient.

A participant talked about his family’s religious beliefs:

*“My family believes that by reading the Quran and praying every daily, evil spirits can be kept away from the house, and by sacrificing sheep and sharing among the poor, God will help the sick to recover sooner” (P.4)*.

## Discussion

In this qualitative content analysis, the experiences of Baloch families with members suffering from schizophrenia were explored in our context. دThe main theme of immersion in the cultural beliefs indicated that the participants believe in the involvement of supernatural factors in the root and treatment of psychological disease. Influenced by cultural ideologies, the Saudi people firmly believe in supernatural powers like the evil eye and supernatural spirits like jinn [11]. In Indonesia also, caregivers believe that the cause of mental illness is supernatural and often turn to traditional healers and shamans for help [29]. If a family believes that the cause is a supernatural or religious factor, then they tend to seek spiritual healing as a cure for the disease. This causes a significant delay in the referral of specialized treatment, and consequently leads to a poor prognosis, increased social burden and higher costs of the disease [41].

Attributing the symptoms of schizophrenic disorder to the possession of jinn and magic, evil spirits and supernatural causes is one of the findings of the present study, which is consistent with the findings of Gupta et al. (2021) in Nepal and Sanghvi et al. (2020) in India [28, 41]. This consistency is due to the cultural similarity of the two countries of Pakistan and India with the people living in Sistan and Balochistan province. In the study of Kilic & Saruc (2015) in Turkey, only one of the mothers suspected that her child was bewitched, and the other participants did not mention supernatural factors [42]. In the studies conducted in European and North American countries, even in the studies conducted in other parts of Iran, no study was found on the belief of family members in supernatural factors as a reason for the psychological disease [37, 43–45]. Unlike some Asian countries who believe in superstitions and religious beliefs about the etiology of schizophrenia, Japanese people do not believe in religion or spirituality in the development of the disease. The Japanese believe more in a nervous personality and weakness of character than in religious and otherworldly issues [46]. This difference is related to the unique cultural structures of Sistan and Balochistan province and superstitious beliefs related to the effects of jinn and magic on life, which are very prominent in the society of this province.

Believing in the supernatural nature of the symptoms and seeking help from prayer writers and clerics was very prominent in this study, and each of the participants had, at least once, referred to such persons to find a cure. Even some participants, although several years have passed since the diagnosis of the disease, said that if they find an address of a healer prayer writer in the future, they will refer to him in the hope of treatment. No study was found in other parts of Iran that is consistent with these findings. The results of this study are proof of the uniqueness of the cultural structure, values, and beliefs of the people of Sistan and Balochistan province.

The understanding and interpretation of diseases in diverse cultures is very different and this leads patients to choose completely different methods for treating diseases. People tend to choose treatment based on services that match their ideas or worldview [47]. In fact, there is a cultural response to the manifestations of schizophrenia in families, which may be the use of herbal medicines at home and consultation with a spiritual person, and application of traditional treatment options is due to their interpretation of the patient’s unusual behaviors [48]. In Africa, treatment choices for mental illness are also influenced by culture and religious beliefs. Non-medical treatments prescribed by traditional or religion-aligned groups for mentally ill patients include herbal, animal, homeopathic, and allopathic components [49]. Some Arab-American Muslims refuse treatment for fear of being called “mad” or “majnoon” in Arabic. Fear of appearing weak makes it more difficult to seek treatment [10]. In Pakistan, imams (traditional spiritual leaders) are often considered as indirect agents of God’s will and facilitators of the recovery process, and they play an essential role in shaping the attitude and response of the family and society towards illness [23]. Studies conducted on African Muslims also showed that most Muslims use traditional medicines as the first treatment for all kinds of mental and emotional problems, and traditional healers treat mental illness due to spiritual and supernatural causes, such as the state of being possessed by jinn, “evil eye” and witchcraft [50, 51]. These traditional healers, who may be sheikhs, dervishes, or elders depending on their geographical location, perform various customs and rituals such as reading the Quran, praying, beating the patient to expel the jinn from his body, etc., for the recovery of the patients [51]. The findings of the study by Azman et al. (2017) showed that the families of psychiatric patients in Malaysia sought help from traditional healers known as Bomoh to treat symptoms, which was a trial and error effort that not only did not improve the symptoms, but also, delayed Medical treatment aggravated the patient’s symptoms [52]. Also, in Islamic countries, especially in Arab and Asian countries, a method called Ruqyah is used to free people from demons, and based on the findings of our study, this method is also used in Sistan and Balochistan province. There are different types of Ruqyah treatment, a common feature of which is the recitation of Quranic verses or prayers, but different Ruqyah treatment centers have different methods, procedures or approaches [53]. Another finding of this study was that some families eventually admitted patients with schizophrenia to the hospital, despite following non-therapeutic treatment ways based on their cultural beliefs. The finding shows that lack of knowledge about this disease and its progression leads to resorting to alternative treatments rather than medical treatment [54]. Although there is a time between the appearance of symptoms and receiving appropriate treatment in these patients, which leads to a delay in treatment [11], in the end, they have to seek therapeutic measures.

### Limitations

The present study was conducted on the Baloch ethnic group living in southeastern Iran, so generalization to other Baloch ethnic groups living in countries such as Afghanistan, Pakistan and others requires further studies of other cultural groups.

## Conclusions

In general, caregivers of patients with schizophrenia are very distressed. On the other hand, living with and caring for patients with schizophrenia without medical and scientific treatment causes extreme suffering. Families are upset and horrified by the patient with regressive symptoms, and worry about the family’s reputation and the stigma caused by the disease as well as the cost of non-medical treatment. Furthermore, the lack of income and financial support, besides the disruption of parental roles turn the family situation into a crisis.

The findings of the present study showed that patients with schizophrenia and family caregivers are influenced by cultural and ethnic beliefs. The occurrence of symptoms, treatment and rehabilitation of these patients are affected by the culture and context in which they live. These beliefs are very prominent in Sistan and Balochistan province, especially in the Baloch ethnicity, which forms an important part of the cultural structure of this province. In this culture, the patients’ psychopathic behaviors are attributed to jinn, magic, evil eye, conspiracy of the people around them, and they seek treatment first through non-therapeutic ways. Theses cultural beliefs and superstitions can cause serious problems in the treatment process of patients with schizophrenia and in the help-seeking behaviors of family caregivers. Culturally and context-based treatments include referring to prayer writers, spell breakers, voiding magic, seeking help from mullahs, and religious approaches. It is concluded that although there is no alternative to scientific treatments for schizophrenia, cultural beliefs cannot be changed easily and in a short period of time. It seems likely that encouraging people to use medical treatments and these methods as suggestive and auxiliary methods may improve the treatment of patients and prevent their dissociation. Thus, psycho-education should be part of all mental health education programs in these communities, as delays in treatment worsen the prognosis of people with schizophrenia. It is recommended that training the medical staff to consider the culture, religion and therapeutic preferences of the Baloch people can be effective in advancing the goals. In addition, local influencers should stress the importance of healthcare alongside harmless local remedies. The role of treatment in controlling the schizophrenia and preventing the deterioration of the patient should be spread among the people through local influential people.

## Data Availability

The datasets generated and analyzed during the current study are not publicly available due to limitations of ethical approval involving the patient data and anonymity, but are available from the corresponding author on reasonable request.
